# Targeting Cytosolic Nucleic Acid-Sensing Pathways for Cancer Immunotherapies

**DOI:** 10.3389/fimmu.2018.00711

**Published:** 2018-04-09

**Authors:** Sandra Iurescia, Daniela Fioretti, Monica Rinaldi

**Affiliations:** Department of Biomedical Sciences, Institute of Translational Pharmacology, National Research Council, Rome, Italy

**Keywords:** DAMPs, STING, RIG-1, innate immune system, cytosolic nucleic acid receptors, antitumor response, agonist, clinical trials

## Abstract

The innate immune system provides the first line of defense against pathogen infection though also influences pathways involved in cancer immunosurveillance. The innate immune system relies on a limited set of germ line-encoded sensors termed pattern recognition receptors (PRRs), signaling proteins and immune response factors. Cytosolic receptors mediate recognition of danger damage-associated molecular patterns (DAMPs) signals. Once activated, these sensors trigger multiple signaling cascades, converging on the production of type I interferons and proinflammatory cytokines. Recent studies revealed that PRRs respond to nucleic acids (NA) released by dying, damaged, cancer cells, as danger DAMPs signals, and presence of signaling proteins across cancer types suggests that these signaling mechanisms may be involved in cancer biology. DAMPs play important roles in shaping adaptive immune responses through the activation of innate immune cells and immunological response to danger DAMPs signals is crucial for the host response to cancer and tumor rejection. Furthermore, PRRs mediate the response to NA in several vaccination strategies, including DNA immunization. As route of double-strand DNA intracellular entry, DNA immunization leads to expression of key components of cytosolic NA-sensing pathways. The involvement of NA-sensing mechanisms in the antitumor response makes these pathways attractive drug targets. Natural and synthetic agonists of NA-sensing pathways can trigger cell death in malignant cells, recruit immune cells, such as DCs, CD8^+^ T cells, and NK cells, into the tumor microenvironment and are being explored as promising adjuvants in cancer immunotherapies. In this minireview, we discuss how cGAS–STING and RIG-I–MAVS pathways have been targeted for cancer treatment in preclinical translational researches. In addition, we present a targeted selection of recent clinical trials employing agonists of cytosolic NA-sensing pathways showing how these pathways are currently being targeted for clinical application in oncology.

## Introduction

The innate immune system provides the first line of defense against pathogen infection. It relies on a small set of germ line-encoded sensors named pattern recognition receptors (PRRs), which are deputized to detection of pathogen-associated molecular patterns (PAMPs) and danger damage-associated molecular patterns (DAMPs) signals.

Nucleic acid (NA)-sensing is an essential mechanism of the innate immunity that utilizes cytosolic receptors to detect extranuclear DNA or extracellular RNA as DAMPs signals (1).

In mammalian cells, two paradigmatic cytosolic NA-sensing pathways are the cyclic GMP-AMP synthase (cGAS)–stimulator of interferon genes (STING) and the RIG-I-like receptors (RLRs)-MAVS pathways, which are responsible for cytosolic DNA and RNA sensing, respectively (2, 3). The cGAS is a DNA sensor protein, which, upon binding double-strand (ds) DNA independently of DNA sequence, and catalyzes the synthesis of 2′-3′-cyclic GMP-AMP (cGAMP) (4). cGAMP functions as a second messenger that, in turn, engages the endoplasmic reticulum (ER)-membrane adaptor protein STING. After its activation STING traffics from the ER via the Golgi to perinuclear endosomes recruiting tank-binding kinase 1 (TBK1). A phosphorylation cascade allows signal transmission leading to activation of interferon regulatory factor (IRF) 3 and nuclear factor κB (NF-κB) that translocate into the nucleus to drive transcription of type-I interferons (IFNs), interferon-stimulated genes (ISGs), proinflammatory cytokines and chemokines (5, 6). Cytosolic dsRNA sensing involves three sensor proteins, namely retinoic acid-induced gene-I (RIG-I), melanoma differentiation-associated gene 5 (MDA5) and laboratory of physiology and genetics 2 (LGP2) (7), collectively referred to as RLRs. RIG-I sensor preferentially detects 5′-triphosphate (5′-3p)-ending RNA and short dsRNA, while MDA5 recognize long dsRNA (8). The signaling pathway proceeds with interaction of RIG-I or MDA5 with the adaptor mitochondrial antiviral-signaling protein (MAVS) located in the outer mitochondrial membrane and activation of IRF3/IRF7 and NF-κB. The activation usually results in IFNs production, consequent induction of ISGs and activation of NF-kB target genes.

**Figure 1:**
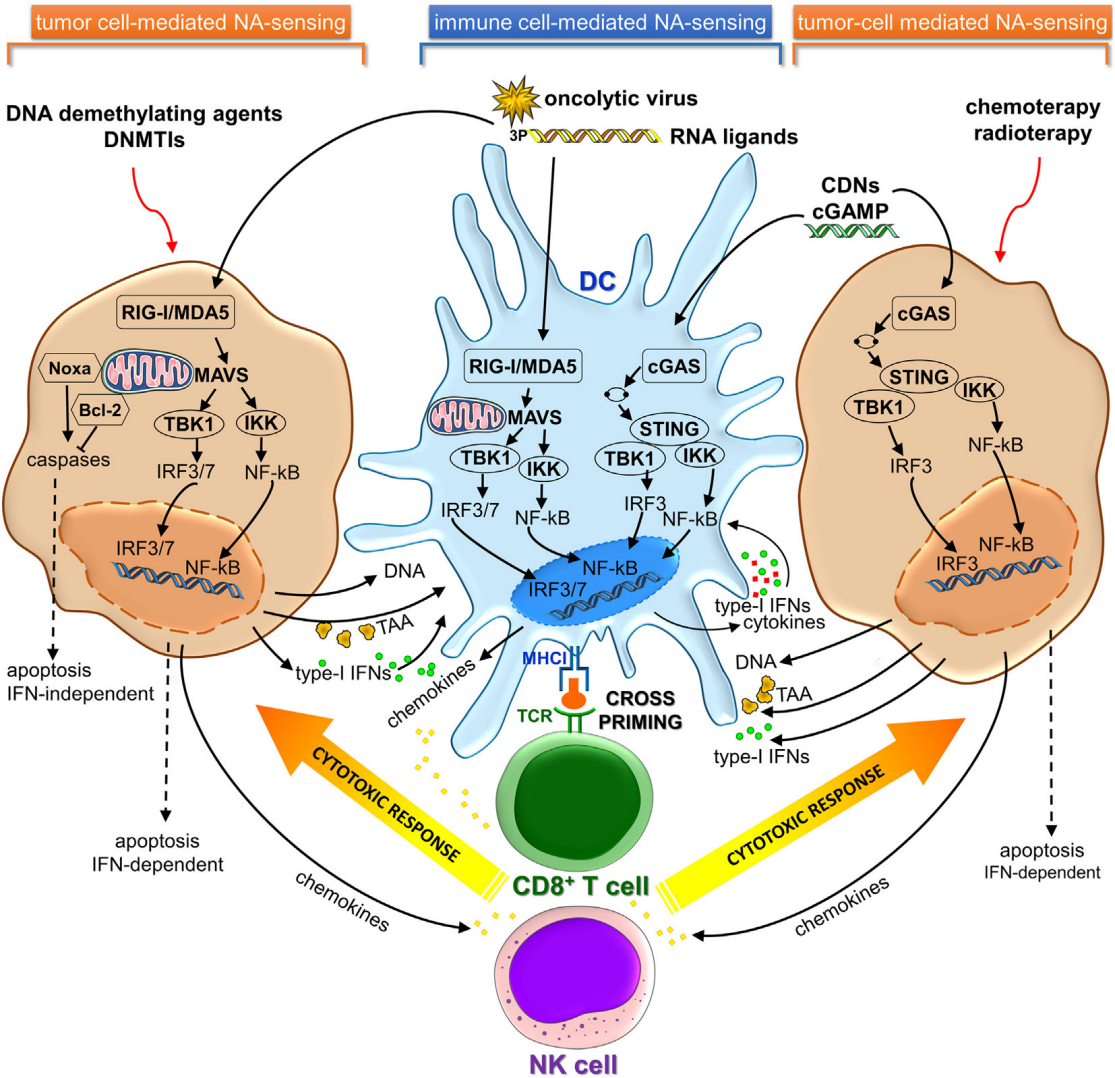
cGAS-STING and RIG-I/MDA5 signaling pathways in immune and cancer cells involved in cancer immunosurveillance and immunotherapy. cGAS, cyclic GMP-AMP synthase; STING, stimulator of interferon genes; RIG-I, retinoic acid-induced gene-I; MDA5, melanoma differentiation-associated gene 5; NA, nucleic acid; TAA, tumor-associated antigen; DNMTIs, DNA methyltransferase inhibitors.

Cancer cells share key hallmarks such as oxidative stress, genome instability and mutations, and altered metabolic rate that can generate nuclear and/or mitochondrial DNA damage (9). Recent studies revealed that damaged NAs released by dying cancer cells can be sensed as DAMP danger signals by PRRs present on CD8α dendritic cells (DCs) in tumor microenvironment (TME), leading to activation of cGAS-STING and/or RIG-I/MDA5 signaling pathways. The consequent type I IFN secretion activates DCs in an autocrine or paracrine manner, resulting in their migration to tumor-draining lymph nodes, where DCs cross-prime naïve CD8^+^ T lymphocytes (10–13) (Figure 1).

Activation of cytosolic DNA sensing pathways impacts on autophagy and tumor antigens (Ags) cross-presentation in DCs. Type-I IFNs production by DCs actually represents the link between NAs sensing and effective Ags cross-presentation to CD8^+^ T cells, therefore linking innate and adaptive immunity (14). Type-I IFNs stimulate upregulation and consequent surface expression of MHC class I genes. Furthermore, type-I IFNs directly promote Ags intracellular retention in DCs that have engulfed apoptotic tumor cells through slowing the endosomal-lysosomal acidification rate, thus enhancing capacity to cross-present Ag by DCs (15–17). Since MHC class I cross-presentation depends on the time of persistence of Ag within the phagolysosomal compartment (16, 18), autophagy possibly provides an intracellular depot where Ag is stored, rather than degraded and represents an alternative pathway for MHC class I presentation (19–21).

Endosomal tumor-derived NAs escape into the DC cytosol through a yet not completely understood mechanism. Specific internalizing receptors such as CLEC9A and CD205 and high-mobility group box 1 protein can mediate uptake of genetic material from dying tumor cells and affect subsequent endosomal trafficking (22–24). Likewise tumor-derived Ags, released DNA could be retained in the endolysosomal compartment where it is preserved before it gains access to the cytosol where it can be recognized by cGAS and other sensor proteins such as intereferon-γ-inducible protein 16 (IFI16), absent in melanoma 2 (AIM2), and Z-DNA-binding protein 1 (ZBP1) (10, 11, 14). The delayed endosomal acidification may further contribute to reduce DNA degradation by DNAse II protease.

Presence of NA sensor proteins across cancer types suggests that these signaling mechanisms may be involved in cancer biology. Actually the expression of RIG-I was significantly downregulated in human hepatic carcinoma (HCC) tissues (25, 26).

Clinical studies revealed that the expression of STING was significantly reduced in HCC tissues compared to the controls, and lower expression of STING was associated with a more advanced tumor stages and a worse survival (27) as well with poor prognosis for patients with gastric cancer (28). Collectively, these studies suggest that STING and RIG-I sensors may serve as tumor suppressors and have clinical values against certain types of tumors as prognostic/predictive biomarkers.

Furthermore, PRRs mediate the response to NAs in several vaccination approaches, including DNA immunization. As route of dsDNA intracellular entry, DNA immunization leads to expression of key components of the cytosolic NA-sensing pathways.

Recent data showed that natural and synthetic agonists of NA-sensing pathways could trigger cell death in malignant cells and recruit immune cells, such as DCs, CD8^+^ T cells, and NK cells into the TME.

In this minireview, we will highlight the newest insights from preclinical studies demonstrating the relevance of manipulating the cGAS-STING and RLRs-MAVS signaling pathways for cancer treatment and how these pathways are currently being targeted pharmacologically (Figure 1).

Clinical evaluation of these innate immune modulators, with agonists alone and in combination with other immunomodulatory agents demonstrates the high translational potential for cGAS-STING and RLRs-MAVS signaling pathway engagement. In Table 1 and Table S1 in Supplementary Material, we present a selection of very recent and novel therapies employing agonists of cytosolic NA-sensing pathways in oncology and provide detailed information concerning mechanisms of action, assessments, and outcomes of reported clinical trials.

**Table 1:** Cytosolic DNA sensors targeting clinical trials.

CT Identifier, Phase Study (Reference)	Trial compound	Condition	Target	Status
NCT02675439, I (29, 30)	MIW815 (ADU-S100)	Advanced/metastatic solid tumors or lymphomas	cGAS-STING pathway	Currently recruiting participantsUpdated on July 2017
NCT03172936, Ib (31)	MIW815 (ADU-S100)/PDR001	Solid tumors and lymphomas		Currently recruiting participantsUpdated November 2017
NCT01274455, I (32, 33)	CYL-02/Gemcitabine	Advanced and/or metastatic and/or non resectable pancreatic adenocarcinoma cancer		Completed Updated on March 2016
NCT02806687, II (34)	CYL-02/Gemcitabine	Advanced, non-metastatic and non-resectable pancreatic adenocarcinoma cancer		Currently recruiting participantsUpdated on February 2017

UMIN000002376, I/II (35–37)	Inactivated Sendai virus particles	Malignant melanoma stage IIIC or stage IV	RIG-I-MAVS signaling pathway	Phase I finished in 2016
UMIN000006142, I/II (38, 39)	Inactivated Sendai virus particles	Castration-resistant prostate cancer		Currently recruiting participantsUpdated on September 2012
NCT01105377, II (40, 41)	Azacitidine/Entinostat	Metastatic colorectal cancer		Completed Update on August 2014
NCT01349959, II (41, 42)	Azacitidine/Entinostat	Advanced breast cancer; triple-negative and hormone-refractory		Ongoing, but not recruiting participantsUpdate on December 2016
NCT01928576, II (43–45)	Azacitidine/Entinostat/Nivolumab	Recurrent metastatic non-small cell lung cancer		Currently recruiting participantsUpdated on October 2017

*CT Identifier, Clinical Trial identifier; RIG-I, retinoic acid-induced gene I; STING, stimulator of interferon genes*.

Results from completed early phase clinical trials with human STING and RIG-I agonists showed biologic and therapeutic effects in patients, leading to combination clinical trials with checkpoint inhibitors (31, 43).

## Preclinical Evaluation of Sting Agonists for Cancer Treatment

Regulation and function of the cGAS-STING pathway has been reviewed elsewhere (3, 46–50), so we will briefly consider newest insights into the topic of STING agonists as potent anticancer agents in preclinical models.

STING pathway has been mostly characterized in APCs, meanwhile in the TME, T cells, endothelial cells, and fibroblasts, stimulated with STING agonists *ex vivo*, have been found to produce type-I IFNs (29). By contrast, most studies indicated that tumor cells developed strategies to inhibit STING pathway activation, likely for immune evasion during carcinogenesis (51, 52).

Recent pieces of evidence have indicated that activation of the STING pathway was correlated to the induction of a spontaneous antitumor T-cell response involving the expression of type-I IFN genes (3, 10, 53). Furthermore, host STING pathway is required for efficient cross-priming of tumor-Ag specific CD8^+^ T cells mediated by DCs (10, 54) (Figure 1).

Based on these findings, direct pharmacologic stimulation of the STING pathway has been explored as a cancer therapy.

Demaria et al. demonstrated CD8^+^ T and type-I IFNs dependent antitumor effect of cGAMP, a natural STING ligand, in melanoma and colon cancer mice models (55).

In 2016, Li et al. confirmed the potent antitumor effect of intratumoral (i.t.) injection of cGAMP in CT26 colon adenocarcinoma-bearing mice. The antitumor activity of cGAMP relied on DC activation and CD8^+^T cell cross-priming (56). More recently, Ohkuri et al. demonstrated accumulation and antitumor effect of potent macrophages in mouse TME of breast cancer, squamous cell carcinomas, colon cancer, and melanoma tissues (57) after i.t. injection of cGAMP.

Canonical cyclic-dinucleotides (CDNs), as direct agonists for STING, show a poor ability to activate human STING. Therefore, an increasing number of synthetic CDNs that potently activate all human STING variants have been designed in recent years (29, 58).

New synthetic CDNs agonist has shown potent antitumor efficiency in various tumor models such as B16F10 melanoma, 4T1 mammary adenocarcinoma, and CT26 colon carcinoma, with regression of established tumor, metastasis rejection, and establishment of long-term immune memory (29).

Recently, many preclinical studies draw a blueprint for the application of STING agonists in tumor therapy in the context of combination therapies.

Strategies that combine STING immunotherapy with other immunomodulatory agents are being explored in mouse models. The antitumor efficacy of cGAMP administered by i.t. injection into B16.F10 tumors was enhanced when combined with anti-programmed death-1 (PD-1) and anti-cytotoxic T-lymphocyte associated-4 (CTLA-4) antibodies (55). In other studies, CDNs together with anti-PD-1 incited much stronger antitumor effects than monotherapy in a mouse model of squamous cell carcinoma model as well of melanoma (59, 60). Luo et al. showed great synergy by combining a STING-activating nanovaccine and an anti-PD1 antibody, and suggested generation of long-term antitumor memory in TC-1 tumor model (61). STING agonists can enhance antitumor responses when combining with tumor vaccines. CDN ligands formulated with granulocyte-macrophage colony-stimulating factor-producing cellular cancer vaccines, termed STINGVAX, showed strong *in vivo* therapeutic efficacy in several models of established cancer. Antitumor activity was STING dependent and corresponded to activation of DCs and tumor antigen-specific CD8^+^ T cells. STINGVAX combined with PD-1 blockade induced regression of poorly immunogenic tumors that did not respond to PD-1 blockade alone (62). STING agonists in combination with traditional chemotherapeutic agents or radiotherapy can work synergistically to trigger antitumor response (56, 63).

The focus of STING pathway agonists for clinical use has thus far centered on their role as vaccine adjuvants and as cancer immunotherapeutic agents for treatment of solid tumors. However, induction of type-I IFNs and other inflammatory cytokines through STING pathway activation results in potent leukemia-specific immunity, culminating in impressive improvements in survival of preclinical acute myeloid leukemia models. Thus, Curran et al. provided solid rationale for clinical translation of STING agonists as immune therapy for leukemia and other hematologic cancers (64).

The intricate STING role may be associated with cell type and activated intensity of downstream signaling. Agonist-mediated activation of STING induces apoptosis in malignant B-cells through specific cytotoxicity, suggesting the potential therapeutic use of STING agonists in treating B-cell malignancies (65). Meanwhile, STING activation reverses lymphoma-mediated resistance to antibody immunotherapy through macrophage activation and modulation of intratumoral macrophage phenotype, as showed by Dahal et al. (66). The induction of apoptosis seems to be a general effector response of the STING pathway in lymphocytes. Gulen et al. reported that overt stimulation of the STING pathway in primary and malignant T cells elicits apoptosis through induction of IRF-3-dependent and p53-dependent proapoptotic genes. This phenomenon, which is evident upon strong stimulus delivery, reveals that the signaling strength determines proapoptotic functions of STING (67). In agreement, low and short *in vivo* activation of STING in T cells provokes type-I IFNs production and ISGs expression mimicking the response of innate cells (68).

## Targeting RIG-I/MDA5 Pathway for Cancer Therapy

RIG-I-like receptors are expressed in most tissues, including cancer cells (69). Recent studies have demonstrated that promising druggable targets against cancer may be represented by components of antiviral immune response. Tumor cells and virus-infected cells can be regarded as injured host cells sharing common features (70, 71). In fact, cancer cells can be induced to mimic a viral infection using RLRs ligands to activate cytosolic RNA-sensing pathway and IFN response (44, 72). This activation also can result in stimulation of cytotoxic immune cells, such as NK and CD8^+^ T cells, which kill cancer cells *via* extrinsic and intrinsic apoptosis (73–75). Consequently, activation of RLRs by using synthetic ligands or oncolytic virus in tumor cells can induce cell death in an IFN-dependent or IFN-independent manner (44, 72–74, 76–78) (Figure 1). Several types of bifunctional small interfering RNAs (siRNAs) with 5′-3p ends conferring a non-self RNA PAMP (79) were developed for both silencing oncogenic or immunosuppressive genes and inducing cell death mediated by viral mimicry (12, 13, 73, 77, 80, 81). Systemic administration of a siRNA designed to trigger RIG-I and silence *Bcl2* induced DC-dependent production of IFNs and strongly inhibited tumor growth in B16 melanoma model. These RIG-I-mediated immune responses synergized with siRNA-mediated *Bcl2* silencing to promote massive tumor apoptosis in lung metastases *in vivo* (73). Likewise, in human drug-resistant leukemia cell lines treatment with multifunctional 5′-3P-siRNA downregulated multi-drug resistance 1 (MDR1) expression and triggered RIG-I-dependent intrinsic apoptosis pathway involving upregulation of Noxa protein, cytochrome-*C*, and effector caspases (81). On the other hand, small endogenous non-coding RNAs gave rise to RIG-I:RNA complexes and initiated downstream signaling events, after ionizing radiation treatment (82). Antitumor DNA-demethylating agent, 5-AZA-CdR, and DNA methyltransferase inhibitors (DNMTis) triggered cytosolic sensing of dsRNA in cancer cells activating endogenous retroviruses and, thus, mimicking a viral infection. The increased viral defense gene expression induced a type-I IFN signaling and apoptosis (44, 72). Furthermore, DNMTi treatment potentiated anti-CTLA-4 immune checkpoint therapy in a pre-clinical melanoma model (44).

Besides the dual tumoricidal property, there are several advantages of targeting RIG-I/MDA5 signaling pathway for cancer immunotherapy. It has been reported that malignant cells are highly sensitive to RIG-I/MDA-5 proapoptotic signaling pathway (74, 83), whereas normal cells are less susceptible as they are rescued from apoptosis by upregulation or activation of endogenous *Bcl-xL*, which prevents RIG-I/MDA5-induced cell death (74). Furthermore, RIG-I and MDA5 are able to trigger a p53-independent alternative pathway for the induction of proapoptotic *Noxa*. Hence, RIG-I/MDA5-driven apoptosis is not mediated by the tumor suppressor p53 mutational status in cancer cells (74), which strongly contributes to resistance to chemo- and radiotherapy (84).

In Table 1 and Table S1 in Supplementary Material are reported some RIG-I/MDA5 ligands that are being utilized in clinical trials. Overall, triggering RIG-I/MDA5 pathway results in eliciting both immunostimulatory and proapoptotic activity conferring to RIG-I/MDA5 a pivotal role in tumor evasion from immune surveillance. Yet it is noteworthy that stimulation of cancer cells by RIG-I ligands not only cause apoptosis but also enhance DCs Ag cross-priming through type-I IFNs release and upregulation of MHC class I gene expression (12, 75, 76).

## Stimulation of Innate Immune PRRs by DNA Vaccines

In the last decade, several DNA vaccines products have been licensed for animal use demonstrating the wide applications of the DNA-based vaccine, such as Apex^®^-IHN, West Nile-Innovator^®^, and ONCEPT^®^ (85–88). DNA vaccines can induce both humoral and cellular immune responses. When used in humans, however, DNA vaccines suffer from lower immunogenicity profiles (89).

Several studies confirmed that immunogenicity of DNA vaccines is regulated by critical components of the innate immune system *via* plasmid DNA recognition through the STING–TBK1 pathway.

The DNA vaccine “adjuvant effect” is not TLR9 dependent, indeed, both TLR9- and MyD88-deficient treated mice mount immune responses comparable to wild-type mice (90). Such immunogenicity leads to the production of type-I IFNs that were found to be crucial for both direct- and indirect-antigen presentation *via* distinct cell types (i.e. DCs and muscle cells, respectively), resulting in the adjuvant effect for the encoded antigen (91, 92). However, the requirement for IFN-αβ in generating high-level antibody responses has yielded contradictory results. The necessity for IRF3 in cellular-mediated immune responses was previously demonstrated, but with a more limited impact, as described by Suschak et al. (90). Indeed, the temporary defect in immune priming provided by *Irf3* deletion is overcome by the induction of *Irf7*, allowing for rescue of DNA vaccine immunogenicity.

The acknowledged versatility of plasmid DNA facilitates the co-delivery of genetic adjuvants encoding immune-stimulatory molecules that need to be overexpressed and selected antigen/s, producing a new generation of vaccines that stand out for their safety and feasibility. In a preclinical study, the co-administration of two plasmid vectors one encoding the DNA sensor DAI and the other one the melanoma-associated antigen tyrosinase-related protein-2 (TRP2) resulted in enhanced tumor rejection and protection against B16 melanoma challenge (93).

The concerted stimulation of innate immune PRRs by DNA vaccines can achieve a more potent and broader activation of the immune responses and a long-lasting protective adaptive immunity.

Co-expressing TBK1 and the serine repeat antigen, a candidate vaccine antigen expressed in the blood-stages of *Plasmodium falciparum*, in the same plasmid backbone enhanced the antigen-specific humoral immune responses, but failed to improve cellular immune responses (91).

Favorable safety profile and potential clinical benefit were achieved after the phase I clinical trial on the activity of CYL-02, a non-viral gene therapy product that sensitizes pancreatic cancer cells to gemcitabine, a chemotherapic acting as a STING pathway agonist, and a non-invasive biomarkers for patient selection was identified (32).

RIG-I and MDA5-activating DNA vaccines can elicit apoptotic and immunostimulatory effects and, thus, could induce growth inhibition or apoptosis of multiple types of cancer cells. Plasmid vector backbones expressing composite immunostimulatory RNAs that act as synergic RIG-I agonists lead to type-I IFNs production (92, 94).

## Conclusion and Perspectives

The spatiotemporal orchestration of innate stimulation with antigen cross-presentation in APCs represents a crucial challenge in reaching a strong tumor specific T-cell response, which in turn is crucial for cancer immunotherapy.

Several studies suggest that cytosolic NA-sensing plays a central role in inducing and bridging innate immunity and adaptive immune responses against tumors and that triggering innate immune system contribute to counteract tumor-induced immunosuppression. Employment of RIG-I/MDA5 and cGAS-STING agonists could represent a novel strategy for cancer immunotherapy.

The role of cGAS-STING and RLRs-MAVS pathways in tumor immunity remains complex and numerous questions still remain unanswered.

Noteworthy, some studies suggest that STING activation may induce a suppressive TME, contributing to tumor growth and metastasis and that STING agonists may not be effective against all tumors, particularly those with tolerogenic responses to DNA and low tumor antigenicity (95, 96). Sensing of DNA by specific innate immune or other cell types and different routes of acute or chronic DNA exposure influence immune responses to DNA.

In head and neck squamous cell carcinoma, dose-dependent activation of RIG-I resulted in divergent effects on cancer cell proliferation. Actually, low dose of dsRNA promoted NF-kB- and Akt-dependent cell proliferation and metastasis (97).

The functional consequences of the cGAS-STING and RIG-I-MAVS pathways regulation/activation in different cells within the TME require deeper characterization. Targeting multiple pathways may be required for efficacious therapeutic responses in some patients and the crosstalk between RIG-I and STING pathways through direct interactions between downstream signaling components may amplify the innate response.

The complex role of STING and RIG-I signaling in cancer underlines how innate immune pathways in the TME alter tumorigenesis in distinct tumors, with effects in designing efficacious immunotherapy trials.

## Author Contributions

SI: searched for literature articles, conceived and wrote the manuscript, conceived the figure, and revised and approved the final version of the manuscript. DF: performed search for clinical trials, collected and analyzed clinical trials data, prepared the tables, conceived and prepared the figure, contributed to the manuscript preparation and editing, and revised and approved the final version of the manuscript. MR: searched for literature articles, conceived and wrote the manuscript, edited the manuscript, and revised and approved the final version of the manuscript.

## Conflict of Interest Statement

The authors declare that the research was conducted in the absence of any commercial or financial relationships that could be construed as a potential conflict of interest.
