# Supplementary material for: Targeting Cytosolic Nucleic Acid-Sensing Pathways for Cancer Immunotherapies
Source: Front Immunol. 2018 Apr 9;9:711. doi: 10.3389/fimmu.2018.00711 (PMC5900005; doi:10.3389/fimmu.2018.00711)
Supplement: Supplementary file 1 [file table_1.PDF]

Supplementary Material

Targeting cytosolic nucleic acid-sensing pathways for cancer immunotherapies

Sandra Iurescia[1≠*], Daniela Fioretti[1≠] and Monica Rinaldi[1*]

[1]Institute of Translational Pharmacology, Department of Biomedical Sciences, National Research Council, Rome, Italy

[≠] These authors contributed equally to this work and shared first authorship

***Correspondence:**

Dr. Monica Rinaldi: monica.rinaldi@ift.cnr.it; Dr. Sandra Iurescia: sandra.iurescia@ift.cnr.it

1 Supplementary Figures and Tables

Supplementary Table 1 Cytosolic DNA sensor targeting clinical trials: full data

**CT Identifier**<br>**Phase Study**<br>***Type***<br>**Sponsor**<br>**(References)**	**Trial compound**<br>**Chemical Composition**	**Condition**<br>**Assessment**	**Mechanism**	**Status**<br>**Results**
**NCT02675439**<br><br>I<br><br>*Interventional*<br><br>Aduro Biotech, Inc.<br><br>(29, 30)	**MIW815 (ADU-S100)**<br><br>Synthetic cyclic dinucleotides	Advanced/metastatic solid tumors or lymphomas.<br><br>Administered by intratumoural injection to characterize the safety, tolerability, pharmacokinetics, pharmacodynamics and antitumor activity.	STING pathway agonists. Intratumoral injection of synthetic cyclic dinucleotides targets and activates human STING, which in turn activate tumor-resident DCs by IFN-β induction, to lead to a tumor-specific immune response.	This study is currently recruiting participants. Updated on July 2017.<br><br>/

**NCT03172936**<br>Ib<br>*Interventional*<br>Novartis Pharmaceuticals<br>(31)	**MIW815 (ADU-S100)/PDR001**<br>Synthetic cyclic dinucleotides +anti-PD-1 antibody	Solid Tumors and Lymphomas.<br>Intratumoral injection of MIW815 in combination with a fixed intravenous dose of PDR001 to assess safety, tolerability, pharmacokinetics, pharmacodynamics and antitumor activity.	STING pathway agonists.<br>Combining MIW815 with PD-1 blockade enhances treated and distal antitumor regression and increases the percentage and function of anti-tumor CD8+ T cells in the distal tumor.	This study is currently recruiting participants. Updated November 2017.<br>/
**NCT01274455**<br>I<br>*Interventional*<br>University Hospital, Toulouse<br>(32, 33)	**CYL-02/Gemcitabine**<br>plasmid DNA/PEI encoding somatostatin receptor subtype 2 and deoxycytidine kinase/uridylmonophosphate kinase+Gemcitabine	Advanced and/or metastatic and/or non resectable pancreatic adenocarcinoma cancer.<br>Administered by intratumoural (plasmid) and in vein (Gemcitabine) injections to evaluate the feasibility, tolerance and antitumor effect of repeated intratumoral injection of a gene therapy product (with antitumor and chemo sensitizing effects) combined with gemcitabine.	STING pathway agonists.<br>Plasmid complexes induce an antitumour bystander effect and render gemcitabine treatment more efficient.	This study has been completed. Updated on March 2016.<br>The gene-therapy product CYL-02 is expressed in PDAC tumors (with long-lasting expression within tumor tissues), is distributed within the bloodstream in some extent.<br>When combined with Gemcitabine it can inhibit primary-tumor progression and dissemination.
**NCT02806687**<br>II<br>*Interventional*<br>University Hospital, Toulouse	**CYL-02/Gemcitabine**<br>plasmid DNA/PEI encoding somatostatin receptor subtype 2 and deoxycytidine kinase/uridylmonoph	Advanced, non-metastatic and non-resectable pancreatic adenocarcinoma cancer.<br>To compare the efficacy of intra-tumoral gene delivery of CYL-02 plus		This study is currently recruiting participants. Updated on February 2017.<br>/

(34)	osphate kinase+Gemcitabine	Gemcitabine treatment or Gemcitabine alone		
**UMIN000002376**<br><br>I/II<br><br>*Interventional*<br><br>Department of Dermatology Osaka University Graduate School of Medicine<br><br>(35-37)	**Inactivated Sendai virus particles**<br><br>hemagglutinating virus of Japan envelope (HVJ-E)	Malignant melanoma stage IIIC or stage IV (AJCC)<br><br>Intratumoral administration of inactivated HVJ-E to evaluate safety and tolerability, and to assess the antitumor immunity and validity of inactivated HVJ-E.	Inactivated Sendai virus particles (HVJ-E) selectively upregulates TRAIL and Noxa (RIG-I-MAVS signalling pathway) in cancer cells.<br><br>HVJ-E is able to fuse to adjacent tumor cells and DCs.<br><br>The apoptotic activity is specific.<br><br>The subsequent antitumor immunity is dependent on HVJ-E-fused DCs and immunocompetent cells, rather than on HVJ-E itself.<br><br>Immunological effects of HVJ-E include enhanced effector T cell- and/or natural<br><br>killer (NK) cell-mediated immunity, activation of DCs to produce IL-6, which suppress the<br><br>function of Treg.<br><br>The anti-tumor effect is dose-dependent.	The phase I clinical trial was finished in 2016.<br><br>Assessed safety and tolerability.
**UMIN000006142**<br><br>I/II<br><br>*Interventional*<br><br>Department of Urology, Osaka University Graduate School of Medicine<br><br>(38,39)	**Inactivated Sendai virus particles**<br><br>hemagglutinating virus of Japan envelope (HVJ-E)	Castration-resistant prostate cancer.<br><br>Intratumoral and subcutaneous injection of HVJ-E to assess safety and efficacy.		This study is currently recruiting participants. Updated on September 2012.<br><br>HVJ-E injection into the prostate followed by subcutaneous injection was well tolerated and feasible, and the complete decline of PSA levels was observed in a subgroup of patients with metastatic CRPC.
**NCT01105377**<br><br>II<br><br>*Interventional*	**Azacitidine/Entinostat**<br><br>DNMT inhibitor (5-azacitidine)+HDAC	Metastatic Colorectal Cancer<br><br>Administered subcutaneously	Epigenetic therapy. Azacitidine is a hypomethylating agent. DNMT	This study has been completed. Update on August 2014

National Cancer Institute (NCI)<br><br>(40, 41)	inhibitor (SNDX-275)	(azacitidine) and orally (entinostat):<br>· to determine the efficacy response rate of the drugs combination;<br>· to assess the toxicity for the combination therapy;<br>· to evaluate any epigenetic changes.	inhibitor upregulate immune signaling in cancer through the viral defense pathway. DNMTis trigger cytosolic sensing of dsRNA causing a type I interferon response and apoptosis. Entinostat is a HDAC inhibitor (HDACi) and regulating a significant number of immune genes.	Samples from selected patient biopsies showed upregulation of AZA IMmune gene set (AIMs) genes after treatment with epigenetic therapy.
**NCT01349959**<br><br>II<br><br>*Interventional*<br><br>National Cancer Institute (NCI)<br><br>(41, 42)	**Azacitidine/Entinostat**<br><br>DNMT inhibitor (5-azacitidine)+HDAC inhibitor (SNDX-275)	Advanced breast cancer; triple-negative and hormone-refractory<br><br>Administered subcutaneously (azacitidine) and orally (entinostat):<br>· to determine the efficacy response rate of the drugs combination;<br>· to assess the toxicity for the combination therapy;<br>· to evaluate any epigenetic changes;<br>· to collect safety and toxicity data as well as the feasibility and response rate where hormonal therapy is added.		This study is ongoing, but not recruiting participants. Update on December 2016<br><br>Samples from selected patient biopsies showed upregulation of AZA IMmune gene set (AIMs) genes after treatment with epigenetic therapy.
**NCT01928576**<br><br>II<br><br>*Interventional*	**Azacitidine/Entinostat/Nivolumab**<br><br>DNMT inhibitor (5-azacitidine)+HDAC inhibitor (SNDX-275)+ PD-1 blocking antibody	Recurrent Metastatic Non-Small Cell Lung Cancer<br><br>Administered subcutaneously	Epigenetic therapy combined with blockade of immune checkpoints – in particular the PD-1/PD-L1 pathway – may augment	This study is currently recruiting participants. Updated on October 2017

Sidney Kimmel Comprehensive Cancer Center<br><br>(43-45)		(azacitidine), orally (entinostat) and (nivolumab) for a randomized study of epigenetic therapy with azacitidine/entinostat and nivolumab *vs.* nivolumab alone.	response of NSCLC by shifting the balance between immune activation and immune inhibition, particularly in a subset of NSCLC with low expression of these pathways.<br><br>"Priming" effect of epigenetic therapy.	

CT Identifier, Clinical Trial identifier; PD-1, programmed cell death protein 1; PEI, Polyethylenimine; RIG-I, retinoic acid-induced gene I; STING, stimulator of interferon genes; AJCC, American Joint Committee on Cancer; DNMTis, DNA methyltransferase inhibitors; HDACi, histone deacetylases inhibitor
